# Pemphigus vegetans misdiagnosed as condylomata acuminata: A case report

**DOI:** 10.1002/ccr3.6393

**Published:** 2022-10-17

**Authors:** Amir Hossein Siadat, Reza Moeine, Fariba Iraji, Hamid Galehdari, Reza Shahriarirad

**Affiliations:** ^1^ Department of Dermatology, Skin Diseases and Leishmaniasis Research Center Isfahan University of Medical Sciences Isfahan Iran; ^2^ Department of Dermatology Isfahan University of Medical Sciences Isfahan Iran; ^3^ Thoracic and Vascular Surgery Research Center Shiraz University of Medical Sciences Shiraz Iran

**Keywords:** condylomata acuminata, diagnosis, pemphigus vegetans

## Abstract

Pemphigus vegetans is a rare variant of pemphigus vulgaris, characterized by vegetating lesions primarily in the flexures. A 41‐year‐old male patient presented with pemphigus vegetans highly mimicking condylomata acuminata, which led to mistreatment. Careful analysis of clinical and laboratory findings enabled us to reach a correct diagnosis and successful treatment.

## Graphical abstract

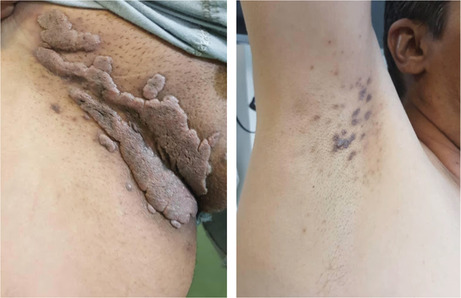

## 1 BACKGROUND

Pemphigus is an autoimmune bullous disorder characterized by autoantibodies to keratinocyte cell surface antigens and is divided into two major forms, pemphigus foliaceus, and pemphigus vulgaris.^1^ Pemphigus vegetans, a variant of pemphigus vulgaris, is characterized clinically by hypertrophic vegetating skin lesions and/or pustules,^1^, ^2^ cauliflower‐like vegetating plaques in the flexures.^3^ The disorder affects chiefly middle‐aged adults and lesions are primarily flexural, although vegetations may occur at any site.^4^ The disease has two main subtypes, the Hallopeau^5^ and Neumann,^6^ in which both have common clinical, immunopathologic, and histologic features, but differ in their prognosis and course.^7^ In the current paper, we report a case of pemphigus vegetans involving the axillary fossae and inguinal area with an unusual presentation that led to misdiagnosis and mistreatment.

## 2 CASE PRESENTATION

**FIGURE 1:**
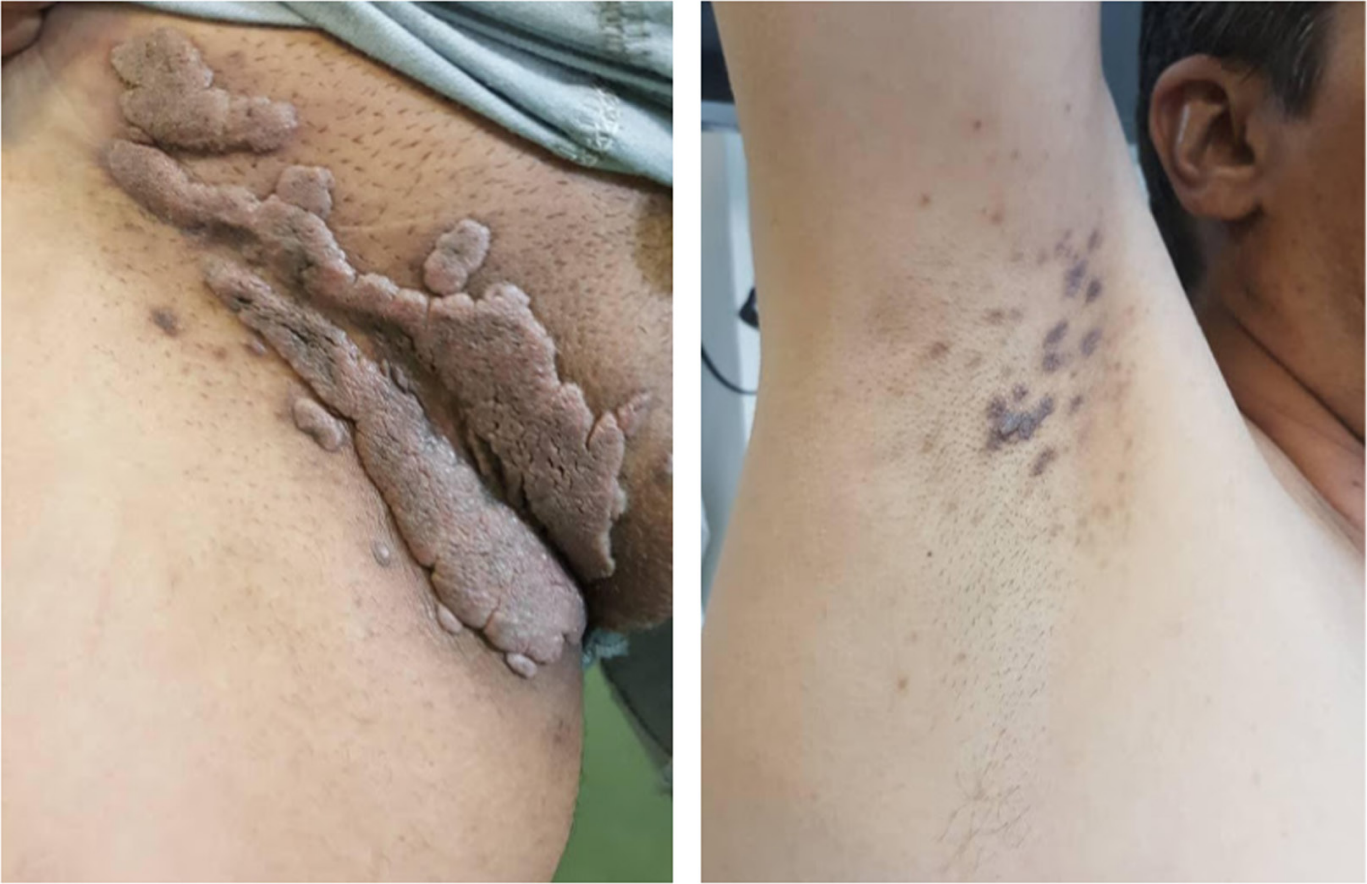
Condyloma‐like lesions in the inguinal area and lichen planus‐like lesions in the axillary area before treatment.

A 41‐year‐old male patient presented with warty lesions to the infectious disease specialist. The onset of lesions was 3 months earlier with the appearance of multiple warty lesions, 1–2.5 cm in size, that were located bilaterally on the groin area (Figure 1). There was no discharge, burning, or itching sensation in the area and the patient denied any pustule or ulcer before their appearance. The lesions were treated with multiple sessions of cryotherapy without any success.

To establish the diagnosis of condylomata acuminata, human papillomavirus (HPV) polymerase chain reaction (PCR) was requested that yielded no evidence of HPV DNA. For a more precise diagnosis, the patient was referred to a dermatologist where body examination showed hyperpigmented, purple macules and papules bilaterally in the axillary area that were also asymptomatic. In addition, oral examination revealed a few, small mucosal erosions that according to the patient, developed after the appearance of the inguinal lesions.

**FIGURE 2:**
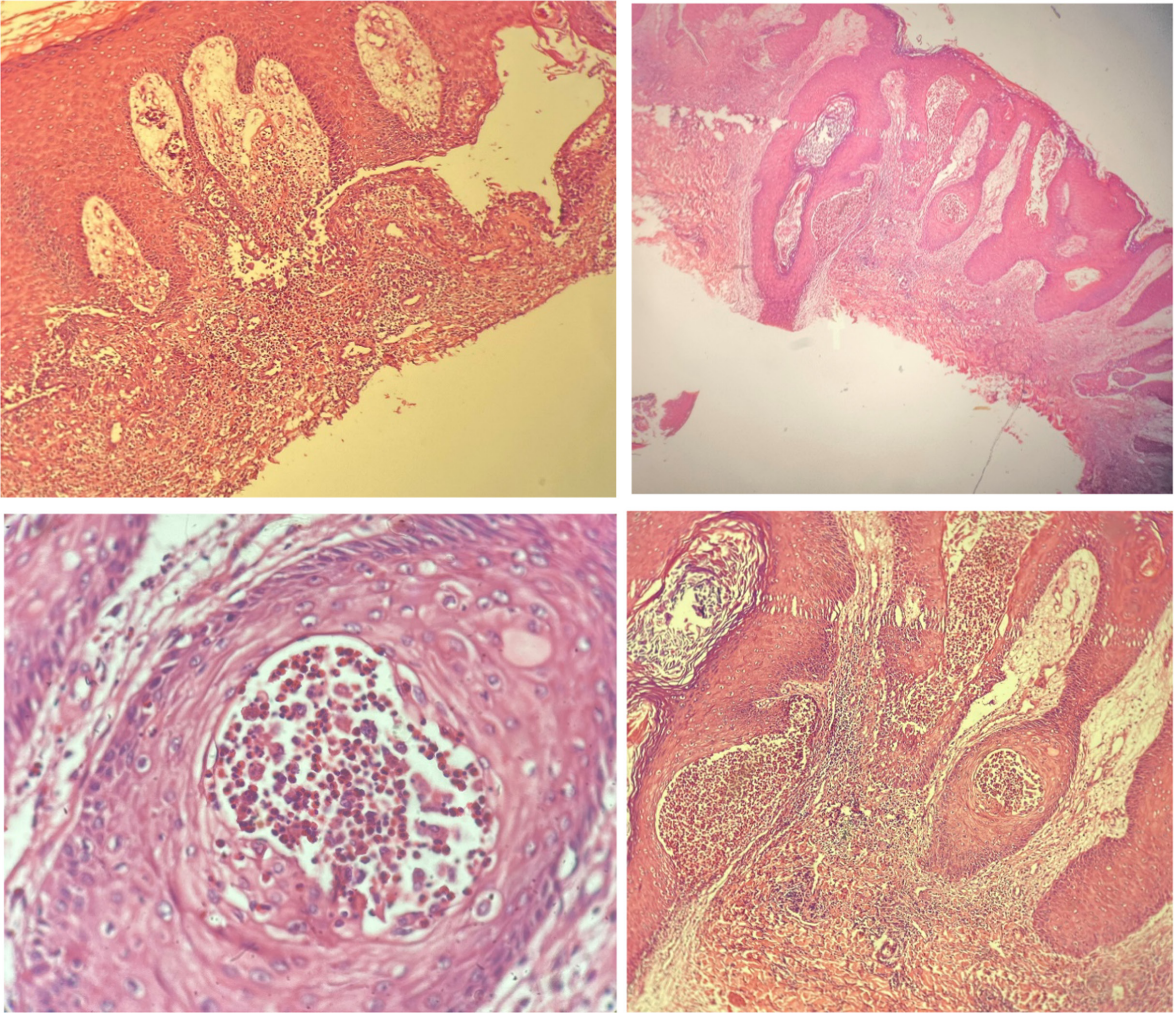
Pathology of the lesion confirming the diagnosis of pemphigus vulgaris.

**FIGURE 3:**
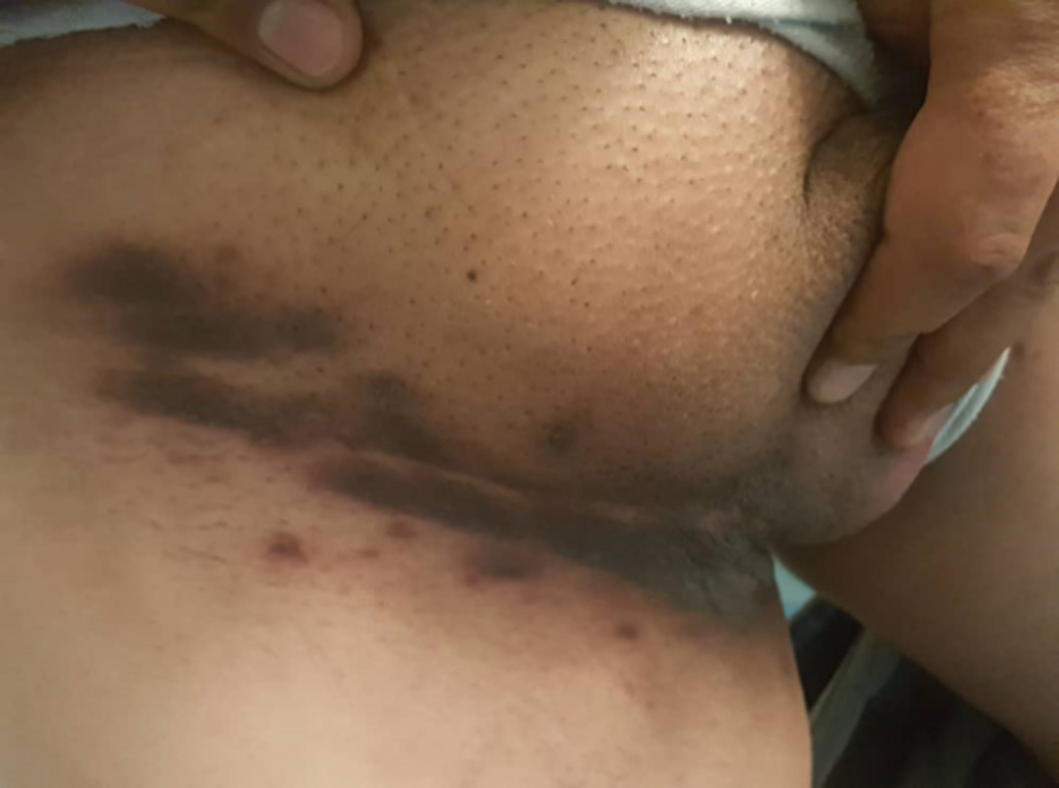
Condyloma‐like lesions in the inguinal area after treatment.

The possible diagnosis of pemphigus vegetans was suggested for the patient and a biopsy sample was obtained from the inguinal verrucous lesions and was evaluated for both pathology and direct immunofluorescence. Pathology evaluation showed focal areas of suprabasal clefts containing a few acantholytic cells along with eosinophils (Figure 2). In addition, hyperkeratosis with focal parakeratosis along with marked acanthosis and spongiosis associated with some eosinophilic microabscess in the epidermis and hair follicles were observed. Direct immunofluorescence evaluation confirmed the intercellular deposition of IgG within the lower epidermis, confirming the diagnosis of pemphigus vegetans. The patient was started on 1 mg/kg of oral prednisolone (60 mg/day), and a dramatic response with almost a complete flattening of the groin and the axillary area was achieved in 2 weeks (Figure 3). The patient is currently under control with a low dose of prednisolone, and there is no recurrence of the lesions.

## 3 DISCUSSION AND CONCLUSION

Pemphigus vegetans is a rare variant of pemphigus. In certain patients, erosions tend to develop into granulation tissue and crusting, known as vegetating lesions,^8^ often found in the groin, armpits, thighs, hands, eyelids, and the perioral region.^9^ Two subtypes Neumann and Hallopeau types are recognized, which are differentiated based on their clinical presentation, course, and response to treatment.^10^, ^11^ Other areas are very uncommonly involved, though reports of lesions limited to foot, oral mucosa, lips, or toes have been described.^11^, ^12^ A lack of familiarity or clinical suspicion may lead to the diagnosis being missed.

In the current case, it was interesting that the patient did not express any complaint regarding his oral lesions (that might be used as a clue for appropriate diagnosis) causing them to be overlooked and leading to mistreatment. In addition, the inguinal lesions were completely asymptomatic and had an appearance that was highly reminiscent of condylomata acuminata. Lesions of the axillary area, on the contrary, had a purplish, polyangular pattern that was reminiscent of inverse lichen planus.

In conclusion, we suggest that complete skin and mucosal examination should be performed on any patient complaining of suspicious warty lesions in the flexural area, and proper diagnostic methods are used for doubtful cases before performing any therapeutic intervention. History of practicing safe sex, presence of vegetating lesions on the groin area only and not on the penis or testes, and family history of autoimmune disorders can be beneficial in securing the diagnosis.

## AUTHOR CONTRIBUTIONS

A.S and R.M. diagnosed the case and carried out the treatment. F.I. and H.H. were major contributors in the case management and data collection. R.S drafted the manuscript. All authors read and approved the final manuscript.

## FUNDING INFORMATION

No financial support was received for this case report.

## CONFLICT OF INTEREST

The authors declare that they have no competing interests.

## ETHICAL APPROVAL

Written informed consent was obtained from the patients in our study. The purpose of this research was completely explained to the patient, and they were assured that their information will be kept confidential by the researcher. The present study was approved by the Medical Ethics Committee of the academy.

## CONSENT

Written informed consent was obtained from the patient for publication of this case report and any accompanying images. A copy of the written consent is available for review by the Editor of this journal.

## Data Availability

All data regarding this study have been reported in the manuscript. Please contact the corresponding author if you are interested in any further information.
